# Expression of Opacity Proteins Interferes with the Transmigration of *Neisseria gonorrhoeae* across Polarized Epithelial Cells

**DOI:** 10.1371/journal.pone.0134342

**Published:** 2015-08-05

**Authors:** Daniel C. Stein, Adriana LeVan, Britney Hardy, Liang-Chun Wang, Lindsey Zimmerman, Wenxia Song

**Affiliations:** Department of Cell Biology & Molecular Genetics, University of Maryland, College Park, Maryland, United States of America; Universite de la Mediterranee, FRANCE

## Abstract

*Neisseria gonorrhoeae* (GC) establishes infection at the mucosal surface of the human genital tract, most of which is lined with polarized epithelial cells. GC can cause localized as well as disseminated infections, leading to various complications. GC constantly change their surface structures via phase and antigenic variation, which has been implicated as a means for GC to establish infection at various anatomic locations of male and female genital tracks. However, the exact contribution of each surface molecule to bacterial infectivity remains elusive due to their phase variation. Using a GC derivative that is genetically devoid of all *opa *genes (MS11∆Opa), this study shows that Opa expression interferes with GC transmigration across polarized human epithelial cells. MS11∆Opa transmigrates across polarized epithelial cells much faster and to a greater extent than MS11Opa+, while adhering at a similar level as MS11Opa+. When MS11Opa+, able to phase vary Opa expression, was inoculated, only those bacteria that turn off Opa expression transmigrate across the polarized epithelial monolayer. Similar to bacteria alone or co-cultured with non-polarized epithelial cells, MS11∆Opa fails to form large microcolonies at the apical surface of polarized epithelial cells. Apical inoculation of MS11Opa+, but not MS11∆Opa, induces the recruitment of the Opa host-cell receptor carcinoembryonic antigen–related cell adhesion molecules (CEACAMs) to the apical junction and the vicinity of bacterial adherent sites. Our results suggest that Opa expression limits gonococcal ability to invade into subepithelial tissues by forming tight interactions with neighboring bacteria and by inducing CEACAM redistribution to cell junctions.

## Introduction

The obligate human pathogen *Neisseria gonorrhoeae* (GC) can infect the urogenital, pharyngeal, and rectal mucosa of men and women. GC expresses a variety of virulence determinants on its surface that have been implicated for the various complications of the infection [1]. These surface molecules play various roles in infection. Pili initiate the interaction of GC with epithelial cells and bring the bacteria physically closer to the epithelial surface by retraction [2, 3]. LOS and Opa are responsible for the intimate interactions of GC with epithelial cells by binding to various receptors on the surface of epithelial cells [4, 5]. These molecules also undergo phase and/or antigenic variation. They are expressed *in vivo*, and phase and/or antigenic variation occurs during natural infection [6–9]. For example, multiple Opas were found to be expressed on GC isolates during experimental urethral infection in men, with the predominant Opa variants differing from the inoculum [6].

The importance of Opa in infection is underscored by the findings that Opa expression increases GC fitness in the female genital tract of mice [10], and that the presence of Opa antibodies in the blood of infected women correlates with a reduced risk of gonococcal salpingitis [11]. Opa mediates bacterial adherence to human epithelial cells and neutrophils [12] through binding to carcinoembryonic antigen-related cellular adhesion molecule (CEACAM) family of receptors [13] as well as heparin sulfate proteoglycans (HSPG) [14]. CEACAMs have regulatory roles in both cell-cell adhesion and cell signaling, by interacting with the actin cytoskeleton, β-catenin, and SH2-containing tyrosine phosphatase 1 (SHP1) [15–17]. There are 11 isoforms of Opa that have different binding capabilities to the various members of the CEACAM family [18]. Opa expression is sufficient to mediate invasion, as Opa expression enables *Escherichia coli* to invade human cervical and endometrial epithelial cell lines [19]. CEACAM-Opa interactions enable GC binding to human granulocytes and epithelial cells in a CEACAM-dependent and opsonin-independent manner, which induces GC entry into host cells [20]. How GC utilize the regulatory functions of CEACAMs in cell-cell adhesion and cell signaling for infection is not well understood. It has been reported previously that GC entry can lead to intracellular killing of the bacteria by phagocytes [21] and epithelial cells [22]. Recent studies show that Opa-CEACAM interactions can induce signaling cascades that lead to NF-κB activation and enhanced intracellular killing activity in neutrophils [23, 24]. These suggest that Opa-mediated invasion into cells may act against GC survival in the host.

GC establish infection at the mucosal epithelia of the human genital track using three interrelated events: colonization on epithelia, invasion into epithelial cells, and dissemination into subepithelial tissues. The mechanism by which each of these events contributes to infection at different anatomic locations and consequent complications remains elusive. GC have been found in subepithelial spaces in organ culture and clinical samples [25, 26], suggesting that GC transmigration across the epithelium occurs *in vivo*. Their ability to transmigrate across the epithelial barrier has also been demonstrated *in vitro* using polarized T84 epithelial cells and urethral organ cultures [27–30]. We have shown that GC transmigration is dependent on the ability of bacteria to weaken the epithelial barrier. The interaction of GC with epithelial cells induces disassembly of apical junction complexes, which seal the paracellular space between neighboring epithelial cells, in epidermal growth factor receptor (EGFR)-dependent manner [31, 32]. The apical junction disassembly compromises the barrier function of the junction against the lateral diffusion between the apical and basolateral membrane, consequently disrupting the structural and functional polarity of the epithelium. However, GC-induced junctional disassembly does not significantly increase the permeability of the apical junction to Lucifer yellow and fluorescein [32]. The loss of cell polarity may alter GC-epithelial interactions and bacterial infectivity. Previous studies have shown that pili phase variation is involved in the transmigration of GC and *Neisseria meningitis* across polarized epithelial cells [33, 34]; however, whether Opa proteins also contribute to this process is unknown.

In this study, we examined the role of Opa expression in gonococcal transmigration across polarized epithelial cells using a GC strain with all *opa* genes deleted. We found that in addition to being incapable of forming large and tight aggregates on the apical surface of polarized epithelial cells, GC lacking Opa exhibit both increased rate and level of transmigration across polarized epithelial cells, even though GC expressing or lacking Opa have similar levels of adherence. Our results demonstrate a negative role for Opa in GC transmigration, which suggests that Opa expression would attenuate the ability of gonococci to invade into subepithelial tissues without significantly affecting their ability to colonize polarized epithelial cells.

## Materials and Methods

### Bacterial strains

*N*. *gonorrhoeae* strain MS11 used in this study was obtained from Dr. Herman Schneider at Walter Reed Army Institute for Research and derived from the same vial used to inoculate human volunteers [35]. GC were maintained on gonococcal medium base (Difco) supplemented with 1% Kellogg’s growth supplement [36] in a 5% CO_2_ incubator at 37°C. Pili and Opa phenotypes were selected using a dissecting microscope. All bacteria used in these studies were piliated, except when indicating otherwise. While we selected piliated bacteria from different strains, our sequencing analysis showed that they expressed different *pilE* variants. MS11∆Opa is a strain that has had all 11 *opa* genes deleted, and MS11OpaH is a derivative of MS11∆Opa that expresses OpaH as a phase lock on state, as we described previously [37].

### Polarization of epithelial cells

Human colonic epithelial cells, T84, were obtained from the American Type Culture Collection (ATCC # CCL-248) and grown in a 1:1 mixture of Dulbecco's modified Eagle's medium and Ham's F12 medium containing 1.2 g/L sodium bicarbonate, 2.5 mM L-glutamine, 15 mM HEPES and 0.5 mM sodium pyruvate, supplemented with 7% fetal bovine serum (Sigma Aldrich, St Louis, MO) and Penicillin/Streptomycin mixture (100 units penicillin and 0.1 mg streptomycin/ml; Sigma-Aldrich). To establish a polarized epithelial monolayer, T84 cells (3 x 10^4^/well) were seeded onto polycarbonate transwell filters (6.5 mm diameter with a pore size of 3 μm; Costar), with culture media replaced every another day. The transepithelial electrical resistance (TEER) was measured with Millicell-ERS2 Volt-Ohm Meter (Millipore), and monolayers were considered polarized when the TEER exceeded 750 Ω/cm^2^.

### Gonococcal adherence and transmigration

Bacteria were cultured on GCK agar for 18 h and inspected using a dissecting microscope to verify their Opa and piliation phenotype. Bacteria were suspended to a concentration of 1 x 10^6^ cells/ml in media consisting of a 1:1 mixture of DMEM /Hams F12 medium supplemented with 5% fetal bovine serum and 0.5% Kellogg's growth supplement [36]. Polarized T84 cells were incubated apically with bacteria (1 x 10^5^/well, MOI ~ 10) at 37°C in 5% CO_2_ for various time periods. The media from the apical and basolateral chambers were collected. Bacteria in the basolateral chamber were enumerated as the number of transmigrated GC. The number of cell-associated bacteria was determined by lysing epithelial cells on transwells with 1% saponin for 15 min to release bacteria, and determining the number of bacteria by plating an aliquot on GCK.

### Immunofluorescence microscopy

Immunofluorescence analysis was carried out using a previously published protocol [38]. After apical incubation with GC, epithelial cells on transwells were fixed, permeabilized and stained with antibodies specific for the junctional protein ZO1 (BD Biosciences, San Jose, CA), gonococci [37], and CEACAM1/3/6 (YTH71.3, Santa Cruz Biotechnology, Inc., Dallas, TX), followed by secondary antibodies. Z-series of images of epithelial cells were acquired using a Zeiss 710 confocal fluorescence microscope. The percentage of gonococcal clusters with CEACAM staining patches in the vicinity was determined by visual inspection. The colocalization of CEACAM and ZO1 was quantified by the Pearson correlation coefficients using NIH ImageJ Software. The data were generated from 12 random fields for each condition and three independent experiments.

### Transmission Electron Microscopy

Polarized T84 cells on transwells were incubated with GC in the apical chamber at an MOI of 50:1 for 24 h. Cells were fixed, embedded in Epon812, and sectioned across both the apical and basolateral surfaces. Images were obtained using a Zeiss EM10CA transmission electron microscope.

### Permeability of polarized epithelial monolayer

The epithelial permeability was measured using horseradish peroxidase (HRP) [39] and fluorescein isothiocyanate (FITC). Epithelial cells were incubated apically with HRP (1 μg/ml) or FITC (1 μg/ml) in the presence or absence of bacteria for 6 h. The enzymatic activity of HRP in the apical and basolateral media was determined by its color changing substrate ABTS in 0.2 M sodium citrate containing 0.03% H_2_O_2_ with the absorbance measured at 405 nm. The fluorescence intensity of FITC in the apical and basolateral media was determined at 485 nm using a fluorescent plate reader. The data is presented as the average percentage of HRP or FITC in the basolateral medium relative to the total amount added.

### Statistical analysis

Statistical significance was assessed using the Student’s t-test by Prism software (GraphPad Software).

## Results

### Expression of Opa changes the distribution of GC on the apical surface of polarized epithelial cells

**Fig 1:**
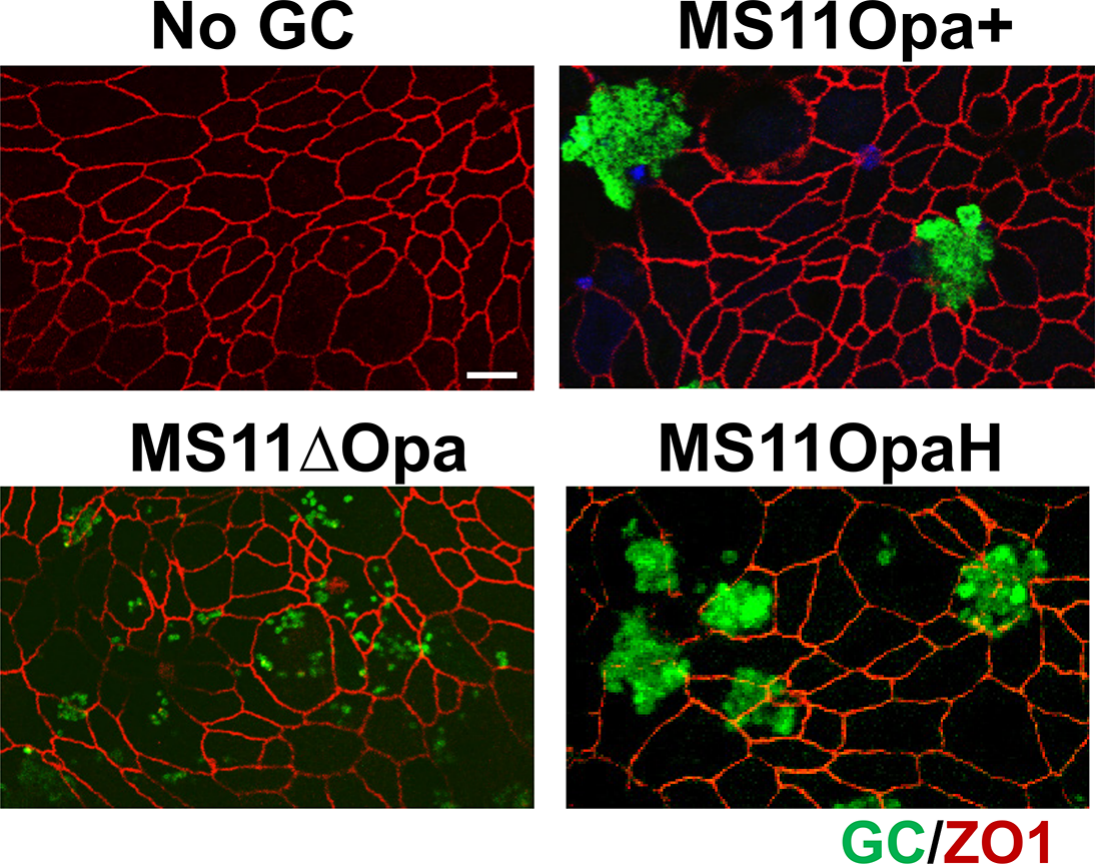
Opa expression changes gonococcal interaction with polarized epithelial cells. Polarized T84 cells on transwells were incubated with or without GC MS11Opa+, MS11ΔOpa, and MS11OpaH for 4 h. Cells were fixed, permeabilized, stained for the junctional protein ZO-1 and gonococci, and analyzed using a confocal microscope. Images shown are representative images from three independent experiments. Scale bar, 5 µm.

We previously demonstrated that Opa expression is critical for GC-GC interactions [37]. MS11Opa+ GC (a strain that can phase vary its Opa expression) but not MS11∆Opa (a strain genetically devoid of all *opa* genes) forms clumps when grown in broth and co-cultured with non-polarized cells. To determine how the expression of Opa impacts gonococcal interactions on mucosal surfaces, we analyzed the distribution of GC on the apical surface of polarized T84 cells grown on transwells. Polarized epithelial cells were incubated with bacteria in the apical chamber at an MOI of ~10 for 4 h. After fixation and extensive washing, cells were stained for the apical junctional protein ZO1 and GC, and examined by confocal fluorescence microscopy. We compared the apical distribution of three GC variants of MS11: MS11Opa+ (Opa-expressing wild type), MS11ΔOpa, and MS11OpaH (a derivative of MS11ΔOpa that had been genetically modified to express OpaH constitutively). We choose to use an OpaH-expressing derivative because this Opa promotes GC invasion into a variety of epithelial cell lines [40, 41]. We found that piliated MS11Opa+ and MS11OpaH formed large microcolonies, while MS11ΔOpa spread across the apical surface of the monolayer, appearing as diplococci or small clumps (Fig 1). While each of these strain express different pilE variants, the pilin that they express all have aspartic acid at residue 140 of the hypervariable region that is associated with low adhesiveness, instead of lysine that is associated with high adhesiveness [42]. These results suggest that Opa is responsible for GC aggregation on the apical surface of polarized epithelial cells, and that GC aggregation can be mediated by the expression of a single isoform of Opa.

### GC interaction regulates the distribution of CEACAMs at the apical surface of polarized epithelial cells

**Fig 2:**
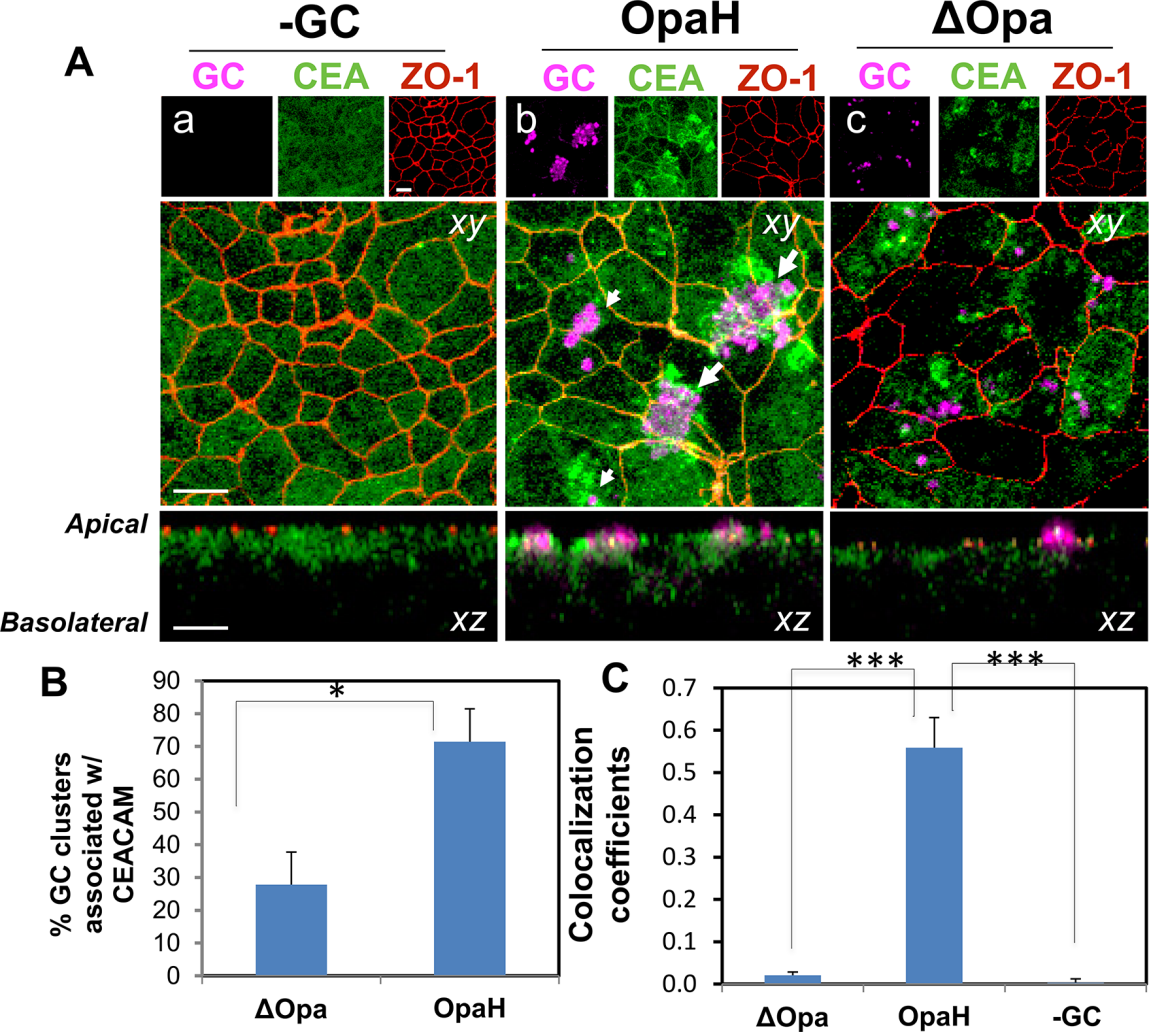
Opa expression alters GC-induced redistribution of CEACAM at the apical surface. Polarized T84 cells on transwells were incubated with GC MS11OpaH and MS11ΔOpa for 4 h. Cells were fixed, permeabilized, stained for the junctional protein ZO-1, gonococci and CEACAM1, and z-series images were acquired using a confocal microscope. Three z-series images at the apical surface were merged (A). Scale bar, 5 µm. Big arrows, big GC aggregates; and small arrows, small GC aggregates. The percentage of GC clusters with CEACAM patches in the vicinity was determined (B). The Pearson correlation coefficient between CEACAM and ZO1 staining was determined using NIH ImageJ software (C). Shown are representative images and the average values from three or four independent experiments. *p = 0.05. ***p = 0.001.

The proteins of the CEACAM family are binding targets of Opa on the surface of epithelial cells. The interaction of Opa with CEACAMs mediates GC adherence to host cells and leads to GC invasion [13, 40]. Therefore, we postulated that Opa-expressing GC but not those without Opa would recruit CEACAMs to adherent sites on polarized epithelial cells. To test this hypothesis, we examined the distribution of CEACAMs at the apical surface of polarized epithelial cells inoculated with or without piliated MS11Opa+, MS11ΔOpa and MS11OpaH using 3D immunofluorescence microscopy. We utilized the immunostaining of ZO1 to mark the apical junction, which divides the apical and basolateral surfaces. In uninoculated T84 cells, CEACAM staining concentrated primarily and distributed evenly at the apical surface (Fig 2Aa). Apical inoculation of either MS11OpaH or MS11ΔOpa induced the redistribution of CEACAMs at the apical surface into irregular patches; however, only in MS11OpaH-inoculated epithelial cells, CEACAM patches were located in the vicinity of bacteria, no matter if they were in big (big arrows) or small (small arrows) aggregates (Fig 2Ab and 2Ac). To evaluate the relationship between CEACAM patches and GC clusters, we determined the percentage of GC clusters with CEACAM patches in the vicinity (Fig 2B). Our results showed that there was a significant higher percentage (71%) of OpaH-expressing GC clusters with CEACAM patches in their vicinity than MS11ΔOpa (28%) (Fig 2B). Furthermore, in MS11OpaH inoculated but not MS11ΔOpa inoculated epithelial cells, CEACAM staining also appeared at the apical junction marked by ZO1 staining (Fig 2Ab and 2Ac). We quantified colocalization of CEACAMs with the apical junction by measuring the Pearson correlation coefficients between CEACAM and ZO1 staining (Fig 2C). We found that there was a significant colocalization between CEACAM and ZO1 staining in MS11OpaH-inoculated cells, compared to no significant CEACAM and ZO1 colocalization in MS11ΔOpa-inoculated and uninoculated epithelial cells (Fig 2C). These results indicate that Opa expression is required for recruitment of CEACAMs to the vicinity of GC adherent sites and the apical junction, but is not essential for the induction of CEACAM redistribution at the apical surface of polarized epithelial cells.

### Opa expression inhibits gonococcal transmigration across polarized T84 monolayers

**Fig 3:**
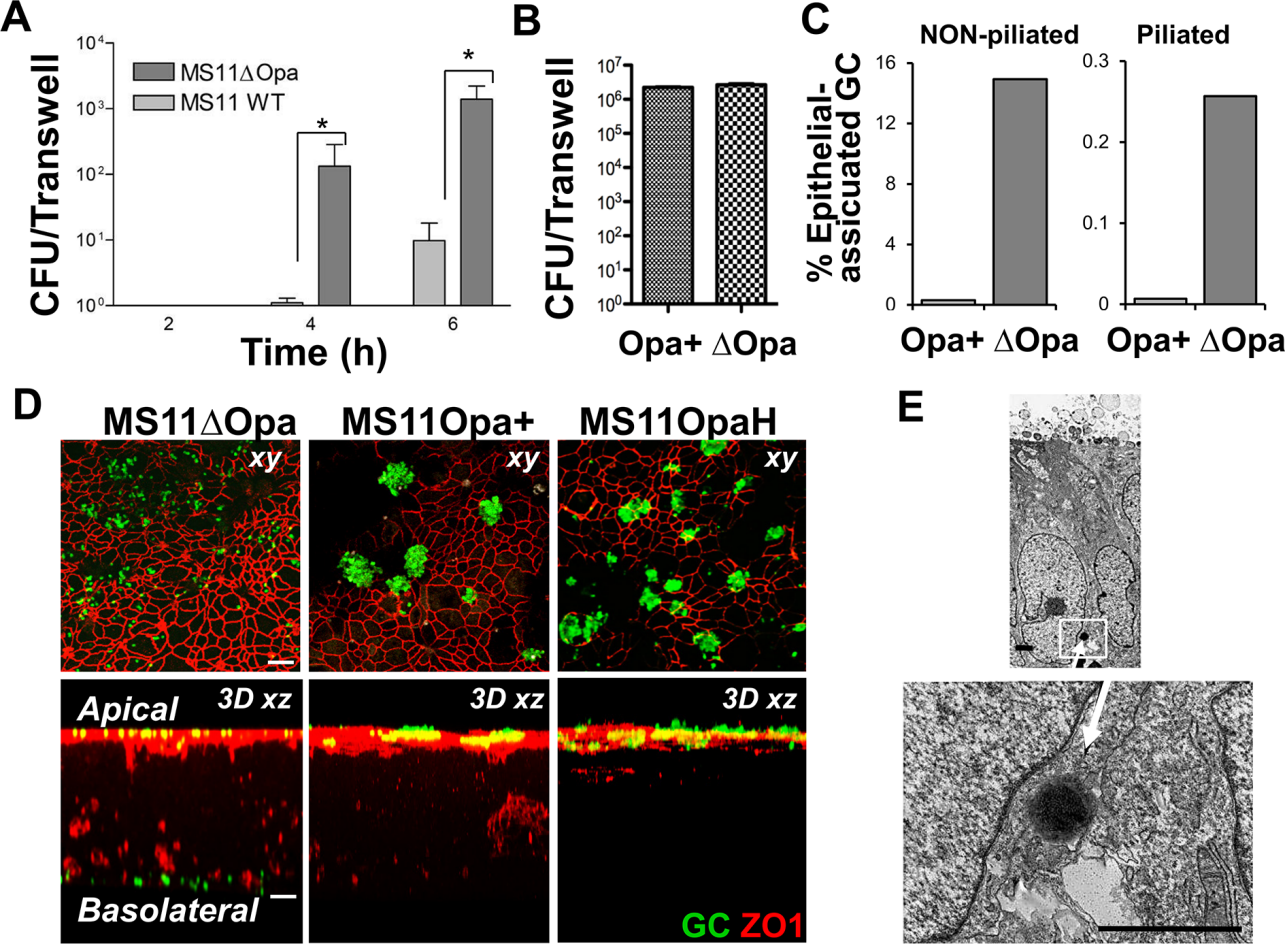
Opa expression reduces the kinetics and magnitude of GC transmigration. (A-C) Polarized T84 cells grown on transwells were incubated with GC apically at a MOI of 10:1 for varying periods of times. The basolateral media were plated onto GCK to enumerate the number of GC that transmigrated into the basolateral media (A and C). Epithelial cells were lysed and plated to determine the number of adhered GC (B). Shown is the average CFU per transwell (A-B) or the average percentage of transmigrated GC over total epithelial-associated GC (C) from three independent experiments, each of which performed in triplicate. *p< 0.05. (D) The cells were fixed and stained for GC and ZO1. Images of z-series were acquired using a confocal microscope. Shown are xy images at the apical junction areas (top) and xz images from 3D reconstitution across both apical and basolateral surfaces (bottom) from three independent experiments. Scale bar, 10 μm. (E) The cells were processed for transmission electronic microscopy. Arrows, GC. Scale bar, 2 μm.

As Opa expression alters the distribution of bacteria and CEACAM at the apical surface of polarized epithelial cells, we investigated whether Opa expression has any impact on the ability of GC to adhere and transmigrate across a polarized epithelial monolayer. Polarized T84 cells were incubated with piliated bacteria in the apical chamber for various times and the number of transmigrated bacteria in the basolateral medium and adhered bacteria in epithelial lysates were enumerated. We were able to collect ~100 CFU of MS11ΔOpa GC in the basolateral media as early as 4 h, compared to an occasional MS11Opa+ (Fig 3A). By 6 h, the basolateral MS11ΔOpa reached >1000 CFU, compared to only ~10 CFU of MS11Opa+ GC in the basolateral medium (Fig 3A). The transmigration efficiency of MS11OpaH was similar to that of MS11Opa+ (data not shown). In contrast, we did not detect a significant difference in the numbers of the two different strains of bacteria adhered to polarized epithelial cells after 6 h incubation (Fig 3B). Furthermore, non-piliated MS11ΔOpa showed a similar increase in the transmigration efficiency over non-piliated MS11Opa+ bacteria (Fig 3C). The similar levels of adherence between MS11Opa+ and MS11ΔOpa and similar increases in the transmigration efficiency of non-piliated MS11ΔOpa over non-piliated MS11Opa+ GC suggest that pili phase variation has no significant impact on GC adherence to and transmigration across polarized epithelial cells. Since Opa deletion does not change the level of GC adherence, this indicates that the different aggregative ability of the Opa+ and ΔOpa strains does not significantly affect bacterial quantification.

The smaller number of Opa-expressing GC in the basolateral medium could be the result of their tight adherence with each other and to the basolateral surface of epithelial cells rather than their decreased ability of transmigration. To test this possibility, we examined the basolateral area of polarized epithelial cells on transwells for the presence of GC using 3D immunofluorescence microscopy. We could readily detect MS11ΔOpa associated with the basolateral surface, but not MS11Opa+ or MS11OpaH in the basolateral region (Fig 3D). The apparent number of MS11ΔOpa associated with the basolateral surface appeared to be much more than the number of CFU that were enumerated in the basolateral media, indicating that the use of colony counting to determine the rate of transmigration of MS11ΔOpa vastly underestimated the true number of transmigrating bacteria.

We used transmission electron microscopy to examine the location of transmigrating GC. We found individual MS11ΔOpa but no MS11Opa+ GC in the paracellular space between epithelial cells (Fig 3E). Taken in toto, these results indicate that Opa expression inhibits gonococcal transmigration across polarized epithelia.

### Only Opa-negative GC are capable of transmigrating across polarized epithelial cells

**Fig 4:**
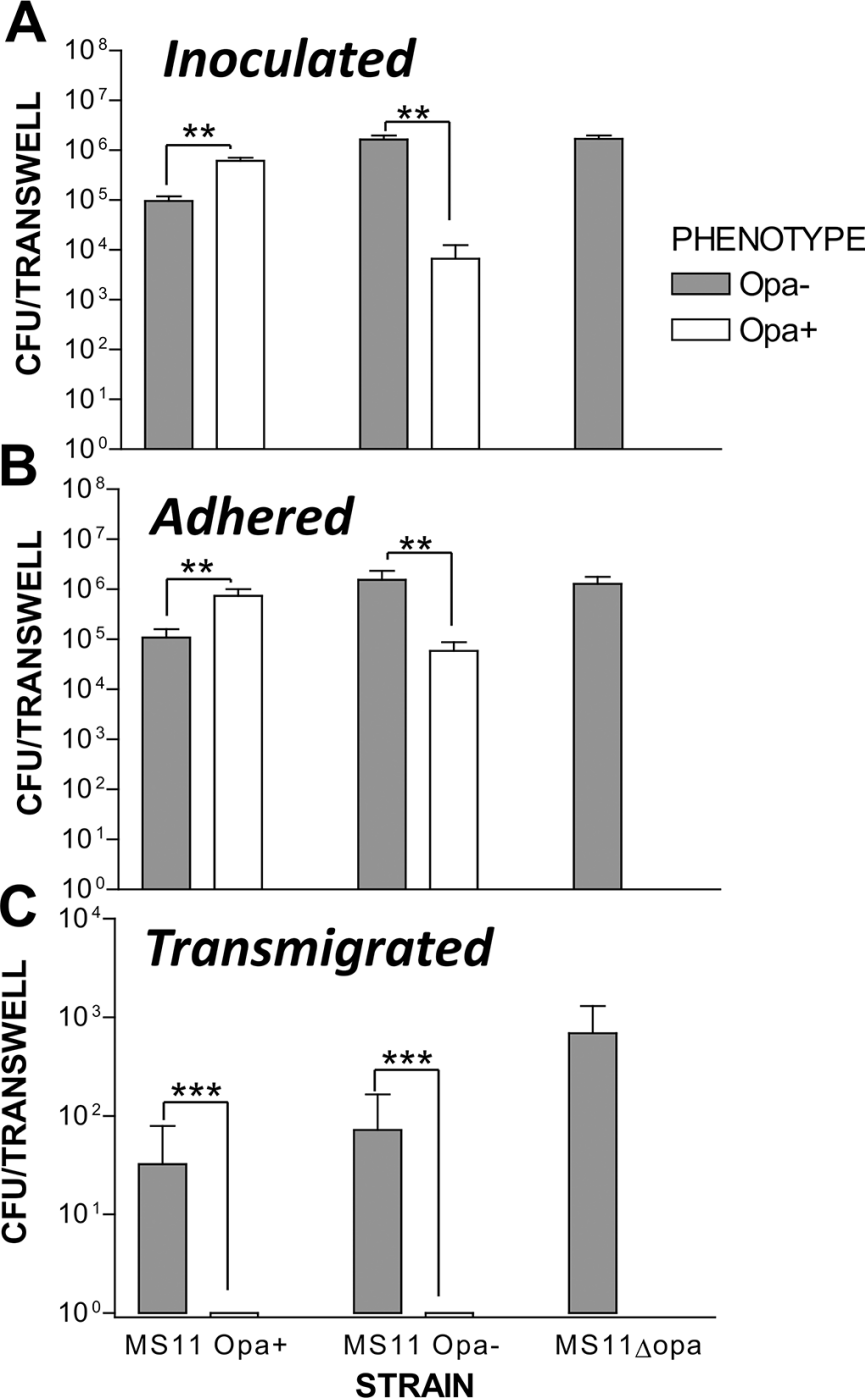
Only Opa- GC can transmigrate across the polarized epithelial monolayer. Polarized T84 cells on transwells were incubated with GC apically at a MOI of 10 for 6 h. The basolateral media were collected. T84 cells were washed and lysed to release cell-associated GC. The inoculum (A), cell lysates (B, adhered) and the basolateral media (C, transmigrated) were culture on GCK plates. CFU were determined. Opa expression of the GC colonies were determined using light microscopy (A and B) or colony blotting based on their binding activity to Mab 4B12. Shown were the average data from three independent experiments each with triplicate samples. **p<0.005, ***p< 0.001.

Our finding that Opa expression decreases GC transmigration across polarized epithelial cells was surprising. To further confirm this result, we determined the number of GC and the Opa expression state of the inoculated (Fig 4A), attached (Fig 4B), and transmigrated GC (Fig 4C) after apical incubation for 6 h with MS11Opa+ (phase variable), MS11Opa- (phenotypically Opa- and phase variable), and MS11ΔOpa. The Opa expression phenotype was determined based on the morphology of resultant bacterial colonies. We found that the Opa phenotype of the attaching GC reflected the phenotype of the inoculum, with ~90% of attached MS11Opa+ strain expressing Opa and ~90% attached MS11Opa-lacking Opa expression (Fig 4A and 4B). In sharp contrast, all transmigrated bacteria were found to be Opa negative even when Opa-expressing MS11 were used to initiate the infection (Fig 4C). These results further confirm that GC that do not express Opa have an advantage over Opa-expressing GC in transmigrating across an epithelial barrier.

### Gonococcal interactions have no effect on the permeability of polarized epithelial monolayers

**Fig 5:**
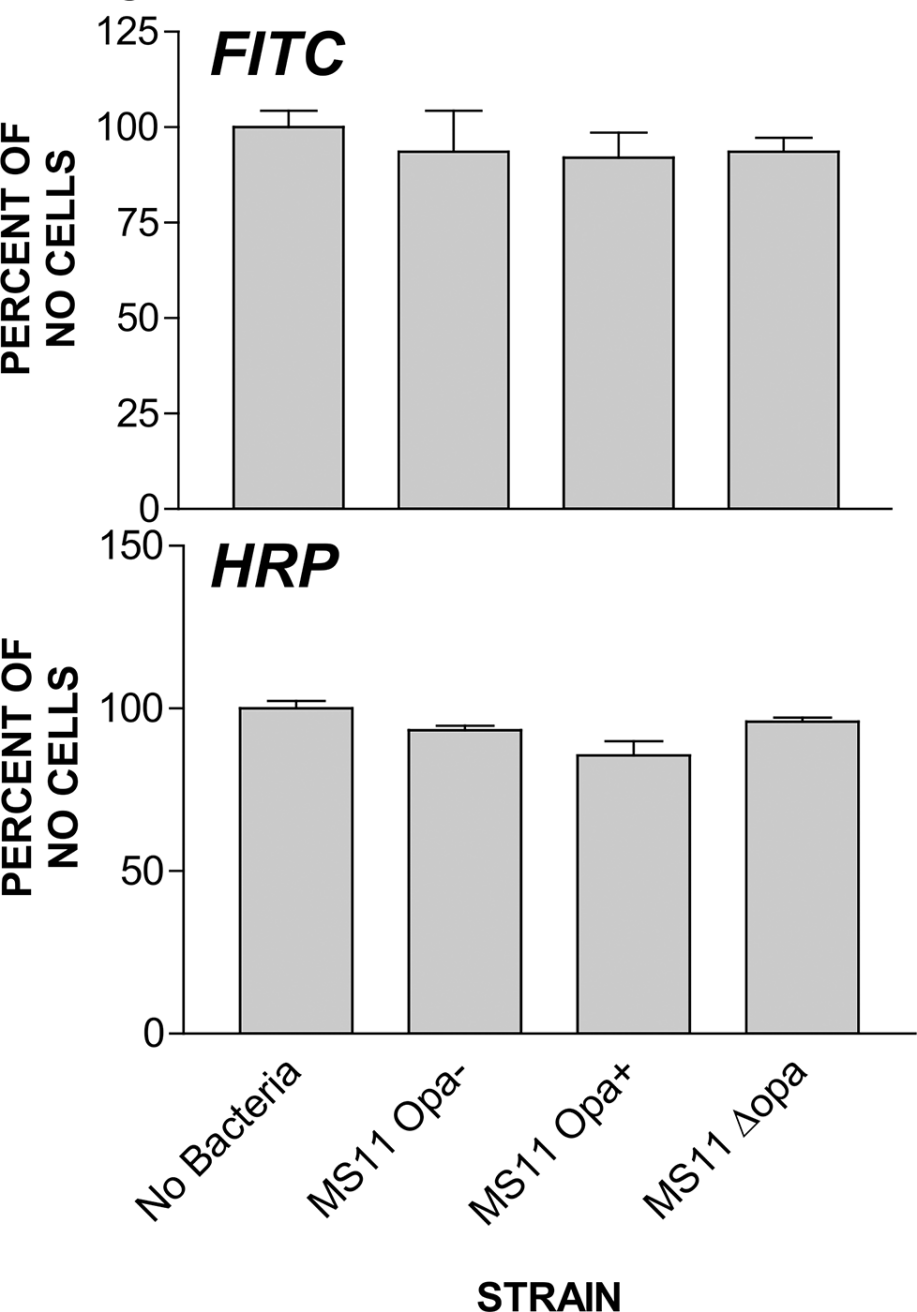
Opa expression has no impact on the permeability of polarized epithelial monolayer infected with GC. Polarized T84 cells were apically incubated with GC for 6 h in the presence of 1 μg/ml of FITC or HRP in the apical medium. The fluorescence intensity of FITC in the basolateral media was measured at 490 nm using a fluorimeter. The enzymatic activity of HRP was measured using a color changing substrate. Shown are the average percent of FITC and HRP leaked into the basolateral media from three independent experiments.

We have previously shown that Opa-expressing MS11 can induce the disassembly of the apical junction. The junction disassembly leads to a reduction in the barrier function of the apical junction against the lateral diffusion between the apical and basolateral membrane, but not a significant increase in the junctional permeability [32]. To determine whether MS11∆Opa GC transmigrate across polarized epithelial cells by making the epithelium paracellulary leaky, we examined the effect of MS11Opa+ and MS11∆Opa inoculation on the permeability of the epithelial monolayer to HRP and FITC. After apical incubation with HRP or FITC for 6 h in the presence or absence of GC, there was no significant increase in the amount of HRP or FITC leaking from the apical to basolateral chamber compared to the control cells without GC inoculation (Fig 5). This result suggests that the relatively high transmigration efficiency of MS11∆Opa is not due to an enhanced ability of MS11∆Opa to increase the permeability of the polarized epithelial monolayer to molecules equal or bigger than FITC and HRP.

## Discussion

This study demonstrates that the expression of Opa, one of the phase variable surface molecules of GC, reduces the ability of this bacterium to transmigrate across polarized epithelial cells. This reduction is associated with the formation of tight and large GC microcolonies on the apical surface of epithelial cells and the redistribution of the Opa host receptors CEACAMs to the apical junction of epithelial cells and the proximity of GC adherent sites. These results suggest that Opa modulates GC infection by regulating GC-GC interaction and the organization and function of its host receptors, CEACAMs.

While the role of Opa in GC infection in various epithelial cell lines has been extensively studied, the cellular mechanisms by which Opa contributes to GC infection and infection outcome remain elusive. By constructing a strain that is genetically devoid of *opa* genes, we demonstrated that Opa expression is required for bacterial-bacterial interactions and the formation of tight and large GC aggregates in suspension cultures and on non-polarized epithelial cells [37]. Using this Opa deletion strain, we extend these observations to polarized epithelia by showing that Opa-mediated bacterial-bacterial interaction sustains when GC interact with the apical surface of polarized epithelial cells. In the absence of Opa, GC fail to form large aggregates, instead spreading as loose small clusters on the apical surface of polarized epithelial cells. The difference in bacterial-bacterial interactions between Opa expressing and lacking GC likely has a direct impact on bacterial interactions with epithelial cells, as fewer bacteria in large aggregates can form direct interactions with epithelial cells than those in small aggregates.

Opa has been shown to be an adhesin, binding to host surface molecules, including CEACAMs and HSPGs. CEACAMs are expressed as different isoforms in various cell types and function as adhesion molecules mediating interactions between host cells [43]. Homophilic and heterophilic interactions of CEACAMs between neighboring cells, which lead to CEACAM oligomerization, induce signaling, regulating cell apoptosis and differentiation [43]. The 11 Opa proteins exhibit various binding affinities to CEACAM isoforms [14, 44], which probably enable GC to bind to different types of host cells. Opa-CEACAM interactions can induce entry of GC into host cells, which may be the results of CEACAM-induced signaling [12]. Our results show that GC interaction induces the redistribution of CEACAMs from homogenous to patches at the apical surface of polarized epithelial cells, implicating the oligomerization of CEACAMs at the cell surface [45, 46]. However, only in epithelial cells inoculated with Opa-expressing GC do CEACAM patches appear in the vicinity of GC adherent sites. However, not all regions of CEACAM patches colocalize with GC aggregates. In addition, inoculating Opa-expressing GC induces the recruitment of CEACAMs to the apical junction. These results suggest that Opa on GC maintains its ability to interact CEACAMs on the apical surface of polarized epithelial cells, and such interactions appear to enhance CEACAM-CEACAM interactions and CEACAM interactions with the apical junction. This would be consistent with the observations of Muenzer et al. [47] who showed that CEACAM-binding GC enhance host cell adhesion. This would have the net effect of strengthening the junctions between epithelial cells [48], thereby restricting the ability of GC to passage between cells. CEACAM-CEACAM interactions have been shown to regulate its signaling function, by modulating the recruitment of SHP-1 [15], which potentially modulates GC-induced signaling in epithelial cells. The finding that ∆Opa GC also trigger the formation of CEACAM patches suggests that in the absence of Opa, GC may be able to regulate CEACAMs indirectly by interacting with other molecules on the surface of epithelial cells, such as pili binding to CR3 [49] and LOS to asialoglycoprotein receptors, or reorganizing membrane domains [5]. While the consequence of such reorganization remains to be determined, such an indirect regulation supports the presence of coordination among GC surface molecules during GC infection.

The major finding of this study is the inhibitory role of Opa in GC transmigration across polarized epithelial cells. The clinical significance of GC invasion into subepithelial tissues remains unclear, due to a lack of an animal model that mimics the later stages of GC infection in humans. However, the presence of bacteria in subepithelial locations in *ex in vivo* studies with Fallopian tubes [50, 51], urethral explants [29] and patient samples [52] suggests that GC transmigration occurs *in vivo*. GC transmigration potentially facilitates bacterial dissemination, colonization at the female reproductive tract during menstruation, and the escape of GC from killing inside epithelial cells.

Polarized epithelial cells provide a physical barrier against pathogen invasion via forming the apical junction complexes along the cell-cell contact. There are two possible ways for GC to overcome this barrier: intracellularly by entering epithelial cells from the apical surface and exiting from the basolateral surface and paracellularly by migrating through the apical junction complex. We have previously demonstrated that GC is capable of compromising the epithelial barrier by inducing the disassembly of the apical junction, which causes an increase in the later diffusion between the apical and basolateral membrane but not in the permeability of the epithelium. Furthermore, the efficiency of GC transmigration is dependent on its ability to weaken the apical junction [32]. This study shows that MS11∆Opa GC increases its transmigration efficiency without changing its ability to adhere to the apical surface or to the permeability of the apical junction to FITC and Lucifer yellow. As Opa is known to mediate GC invasion, these results support the possibility of GC transmigration through the compromised apical junction, but do not exclude the possibility of GC transmigrating intracellularly.

The data from this study suggest several potential mechanisms for Opa to interfere with GC transmigration. First, Opa-LOS mediated bacterial-bacterial interactions, which generate bigger and tighter bacterial aggregates, may physically hinder transmigration. In support of this possibility, we found that GC that have turned off Opa expression, thereby weakening the interaction of these GC with the bacterial microcolony are capable of transmigration. Furthermore, pili, another adhesin that mediate GC-GC interactions, also reduce the transmigration efficiency of GC as well as *Neisseria meningitis* [33, 34]. Second, Opa-induced recruitment of CEACAMs to the apical junction may strengthen the apical junction by interacting with β-catenin and the actin cytoskeleton [17, 53]. Lastly, Opa-CEACAM interaction can regulate the signaling function of CEACAMs, such as their phosphorylation by calmodulin kinase and the binding of SHP-1 to the immunoreceptor tyrosine-based inhibitory motif in their cytoplasmic tail [54]. CEACAM-mediated signaling potentially influences host signaling required for GC transmigration, such as EGFR activation and its downstream signaling. However, the relationship between CEACAM and EGFR signaling is completely unknown and a subject of our future study.

Even though this study mainly focused on Opa, our results do not exclude the role of other surface molecules, such as pili. Pili are responsible for bringing GC physically close to neighboring GC and host cells, facilitating Opa-LOS mediated bacterial-bacterial interaction and Opa-CEACAM mediated bacterial-host cell interaction. In addition, Opa-mediated GC-GC and GC-host cell interactions can be modulated by pili-mediated retraction and Opa-induced signaling by the interaction of pili to their host receptors [55]. Pili have been shown to have a negative role in GC transmigration [33], even though which variant responsible for this negative role is unknown. Because all strains used in this study were pili positive and expressed different pili variants as determined by sequencing analysis, we cannot eliminate the potential role of pili-mediated aggregation.

The inhibitory effect of Opa on GC transmigration found in this study and its role in GC invasion [22, 28, 46] suggest that phase on and off of Opa expression may cause GC to switch infectivity models between invading into epithelial cells and subepithelial tissues. The ability of GC lacking Opa expression to transmigrate rapidly across the epithelium allows them to avoid being killed inside epithelial cells or shed with epithelial cells during menstruation. While Opa negative bacteria may be better suited for survival within the host, being able to avoid neutrophil-mediated uptake and killing [23], they are probably less fit on mucosal surfaces as Opa expression is selected for *in vivo* [6]. These findings provide an explanation for the association of GC phase variation with a variety of responses and clinical manifestations in infected individuals, ranging from asymptomatic infection to potentially life-threatening disease. Further studies are required to delineate the molecular and cellular mechanisms by which Opa regulates GC infective models, consequently influencing infection outcomes.
